# Monkeypox Virus Surveillance — United States, 2024–2025

**DOI:** 10.15585/mmwr.mm7532a1

**Published:** 2026-08-20

**Authors:** Ryan Sandford, Alexandra Tuttle, Leah Aeschleman, Crystal M. Gigante, Whitni Davidson, Kimberly Wilkins, Panayampalli S. Satheshkumar, Agha Ajmal, Christina L. Hutson, Faisal S. Minhaj, Christine M. Hughes

**Affiliations:** ^1^Division of High Consequence Pathogens and Pathology, National Center for Emerging Zoonotic Infectious Diseases, CDC; ^2^Chenega Enterprise Systems and Solutions, LLC, Chesapeake, Virginia.

**Summary**

**What is already known about this topic?**

Clade IIb monkeypox virus (MPXV) has continued to circulate in the United States since a 2022 outbreak.

**What is added by this report?**

The number of reported cases of MPXV infection during September–October 2025 was double that during the same period in 2024, although cases returned to background levels by December 2025. Cases continued to disproportionately affect men who have sex with men. Unvaccinated patients accounted for 76.1% of cases and had 9.7 times the odds of hospitalization, as did those who completed the 2-dose JYNNEOS vaccination series.

**What are the implications for public health practice?**

Persistent low-level transmission of clade IIb MPXV in the United States suggests a likely transition toward endemic circulation. CDC recommends that persons at risk for MPXV exposure complete the recommended 2-dose JYNNEOS vaccination series.

## Abstract

Since the onset of a global outbreak of monkeypox virus (MPXV) infections in 2022, cases have declined substantially in the United States; however, ongoing low-level transmission has persisted. To assess trends in cases of MPXV infection in the United States during 2024–2025, investigators analyzed data from the One CDC Data Platform and the National Notifiable Diseases Surveillance System. In 2024, a total of 2,803 cases of MPXV infection were reported in the United States, including one travel-associated clade Ib case. In 2025, a total of 2,519 cases were reported, including 10 clade Ib cases. The number of reported cases of MPXV infection during September–October 2025 was double that during the same period in 2024, although cases returned to background levels by the end of December 2025. Demographic characteristics of patients with MPXV infections, including distributions by sex, race and ethnicity, sexual orientation, lesion location, and age, were similar to those reported in previous years. Among 860 patients with complete information, the odds of hospitalization among those who were unvaccinated were 9.73 times those who were fully vaccinated when controlling for age group and HIV status. Encouraging completion of the recommended 2-dose vaccination series for persons at risk for MPXV exposure, including travelers to specific countries with ongoing clade I or clade II transmission, could reduce the risk for severe illness and hospitalization. Continued routine surveillance remains important to limiting future spread, monitoring changes in viral evolution, and focusing vaccination efforts on affected areas.

## Introduction

Monkeypox is a vesicular rash illness caused by monkeypox virus (MPXV), an orthopoxvirus, and is nationally notifiable in the United States. Two MPXV clades (clade I and clade II) are recognized, each with subclades (a and b). Transmission of subclade IIb, which caused a 2022 global outbreak of MPXV infections (*1*), persists at low levels in the United States. Subclade Ib emerged in the Democratic Republic of the Congo (DRC) in late 2023, causing an outbreak that continues to spread across approximately 40 countries and includes travel-associated cases in the United States (Global mpox trends | World Health Organization). Although clade I has historically been associated with a more severe disease and higher case-fatality rate (CFR) (*2*), clade Ib MPXV is associated with a CFR of <0.5% in Central Africa; no deaths have been reported among approximately 150 cases from high-income countries (Global mpox trends | World Health Organization).

Nationally, clade II MPXV infections peaked at approximately 3,000 per week during the 2022 outbreak, then sharply declined and remained stable at fewer than 150 cases per week throughout 2023 (*1*). MPXV infection disproportionately affects persons with HIV infection, as well as gay, bisexual, and other men who have sex with men (MSM) (*1*,*3*). In addition, those with virally unsuppressed HIV or with low CD4 T-cell counts are known to be at increased risk for severe MPXV infection and death (*1*). This report describes national case trends of MPXV infections during 2024–2025.

## Methods

### Data Source

Jurisdictions report probable and confirmed cases of MPXV infection (orthopoxvirus- or MPXV-positive laboratory result) to CDC through the One CDC Data Platform and the National Notifiable Diseases Surveillance System. The short case report form of reportable variables is available online.

The analysis in this report includes provisional data from cases with an epidemiologic date* during January 1, 2024–December 31, 2025. Genomic and epidemiologic data were used to assess subclade spread and outbreak-associated mutations. Vaccination status was categorized as fully vaccinated (receipt of 2 doses of JYNNEOS [modified vaccinia Ankara–Bavarian Nordic] ≥14 days before illness onset, with ≥24 days between first and second doses), partially vaccinated (1 dose of JYNNEOS ≥14 days before illness onset), or unvaccinated (no documented dose received) based on health department records or self-report, depending on jurisdiction.

### Characteristics and Trend Analyses

Descriptive analyses were conducted to summarize demographic, epidemiologic, and clinical characteristics of patients with reported cases overall and by year. Geographic and temporal trends were assessed by jurisdiction and year. Incidence was calculated as cases per 100,000 persons at risk for infection using AtlasPlus data for MSM and other subpopulations. This includes MSM with indications for HIV preexposure prophylaxis (PrEP), MSM living with HIV, and additional subpopulations at risk for infection as previously described.^†^

Multivariable logistic regression was used to evaluate associations between vaccination status and hospitalization among patients with complete data. Analyses were conducted in R (version 4.4.1; R Foundation) and the One CDC Data Platform. This activity was reviewed by CDC, deemed not research, and conducted consistent with applicable federal law and CDC policy.^§^

### Genetic Analyses

MPXV sequences with collection dates during January 1, 2024–December 31, 2025, (807) were downloaded from a public database to investigate viral lineage dynamics during 2024–2025.^¶^ A reduced set of 343 high-quality genomes (at least 180,000 nucleotides long with fewer than 2,000 missing nucleotides) was used for phylogenetic analysis. Genome alignment and quality control were performed as previously described (*4*).

### Use of Artificial Intelligence

ChatGPT (GPT-5.5 Thinking) was used to check for consistency and formatting. The tool was not used to generate scientific content, analyze data, interpret findings, create references, or draft conclusions. No data were entered into ChatGPT or any other artificial intelligence large language model. All output was reviewed and edited before inclusion in this report.

## Results

**FIGURE 1:**
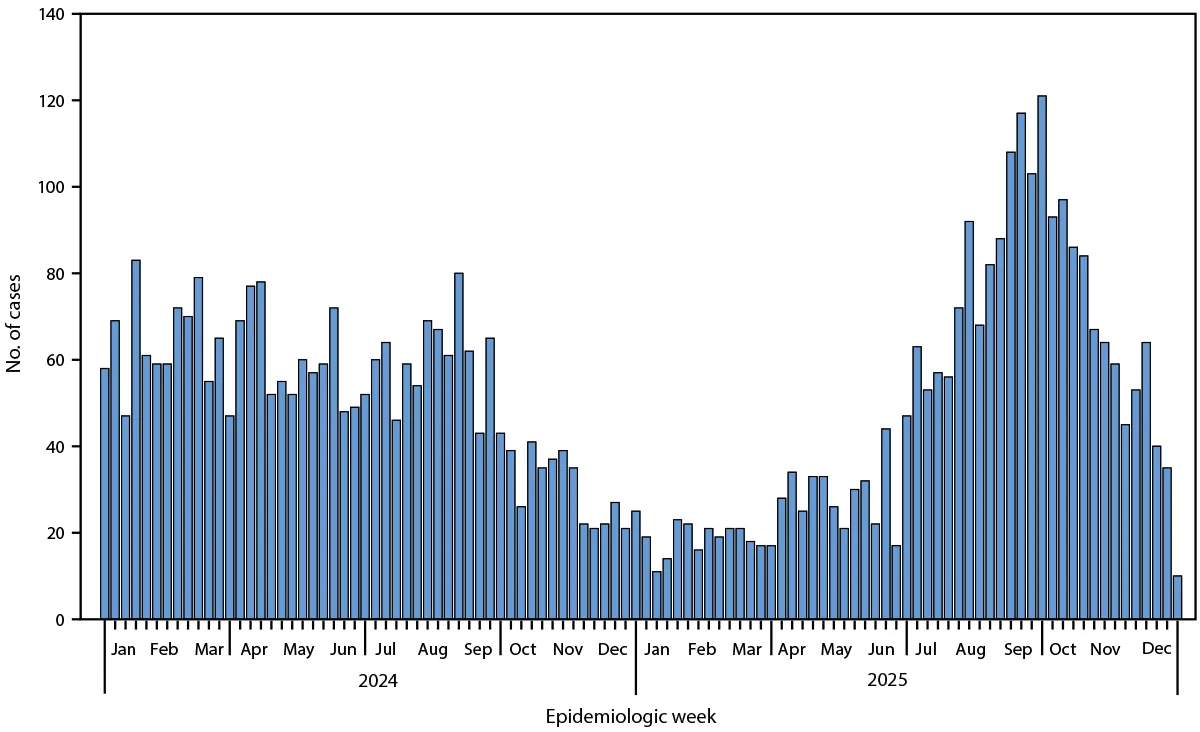
Confirmed* and probable^†^ cases of monkeypox virus infection, by week — United States, 2024–2025 **Abbreviation:** MPXV = monkeypox virus. * Demonstration of the presence of MPXV DNA by polymerase chain reaction testing or next-generation sequencing of a clinical specimen or by isolation of MPXV in culture from a clinical specimen. ^†^ No suspicion of other recent orthopoxvirus exposure (e.g., vaccinia virus in ACAM2000 vaccination) and demonstration of the presence of orthopoxvirus DNA by polymerase chain reaction of a clinical specimen or orthopoxvirus using immunohistochemical or electron microscopy testing methods or demonstration of detectable levels of anti-orthopoxvirus immunoglobulin M antibody during 4–56 days after rash onset.

**FIGURE 2:**
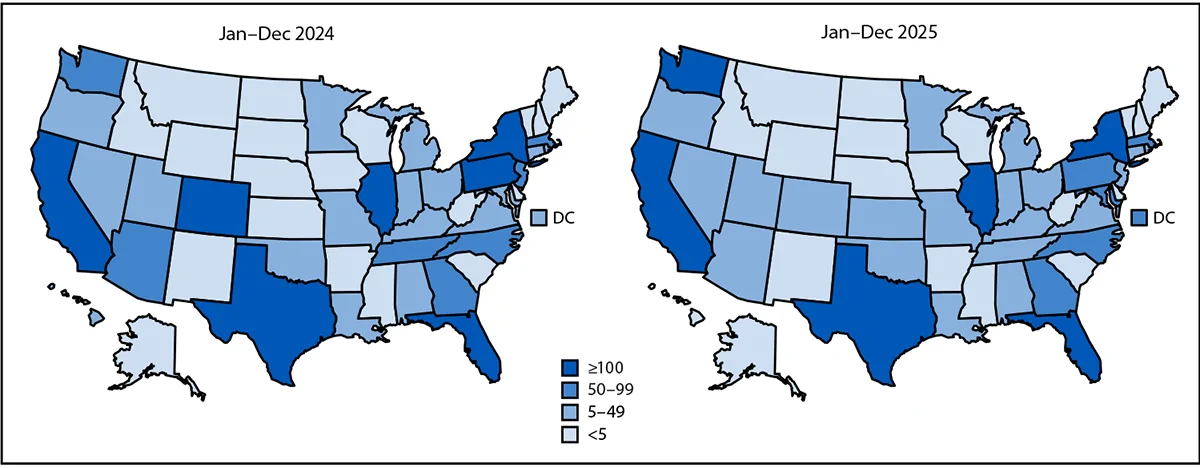
Confirmed* and probable^†^ cases of monkeypox virus infection, by state — United States, 2024 and 2025 **Abbreviations**: DC = District of Columbia; MPXV = monkeypox virus. * Demonstration of the presence of MPXV DNA by polymerase chain reaction testing or next-generation sequencing of a clinical specimen or by isolation of MPXV in culture from a clinical specimen. ^†^ No suspicion of other recent orthopoxvirus exposure (e.g., vaccinia virus in ACAM2000 vaccination) and demonstration of the presence of orthopoxvirus DNA by polymerase chain reaction of a clinical specimen or orthopoxvirus using immunohistochemical or electron microscopy testing methods or by demonstration of detectable levels of anti-orthopoxvirus immunoglobulin M antibody during the period of 4–56 days after rash onset.

During 2024–2025, a total of 5,322 cases of MPXV infection were reported in the United States (Figure 1) (Figure 2). This included 2,803 cases in 2024, including one travel-related case of clade Ib (140 cases per 100,000 population at risk for infection) and 2,519 cases in 2025, including 10 cases of clade Ib (126 per 100,000).

More U.S. counties and municipalities were affected in 2024 (364; 11.3%) than in 2025 (326; 10.1%). The number of reported cases of MPXV infection approximately doubled from 423 during September–October 2024 to 873 during the same period in 2025, before decreasing to 448 cases during November–December 2025.

### Characteristics of Patients with MPXV Infection

Among the 5,322 patients with cases of MPXV infection reported during 2024–2025, sex was reported for 4,613 (86.7%) and age was reported for 5,210 (97.9%). Sex and age distributions were similar in 2024 and 2025. Most cases occurred in males (96.5% in 2024 and 97.8% in 2025), with median patient age of 35 years in 2024 and 36 years in 2025. Among those who identified as male, 79% reported sexual orientation as gay or bisexual. Non-Hispanic Black or African American (22.8%) and Hispanic or Latino (36.3%) patients were disproportionately affected (Table) relative to their representation in the MSM and overall U.S. populations, even though their percentages were lower than those of non-Hispanic White patients.

**TABLE:** Demographic and clinical characteristics of patients with monkeypox virus infection — United States, 2024–2025

Characteristic	No. (%)			p-value*
	2024 n = 2,803	2025 n = 2,519	Total N = 5,322	
**Age group, yrs**				
<18	8 (0.3)	10 (0.4)	**18 (0.3)**	0.25
18–29	780 (28.3)	622 (25.3)	**1,402 (26.9)**	
30–39	1,129 (41.0)	1,068 (43.5)	**2,197 (42.2)**	
40–49	545 (19.8)	530 (21.6)	**1,075 (20.6)**	
50–64	276 (10.0)	211 (8.6)	**487 (9.3)**	
≥65	15 (0.50)	16 (0.7)	**31 (0.6)**	
Missing data^†^	50	62	**112**	
**Age, yrs**				
Mean (SD)	35.8 (9.9)	36.0 (9.5)	**35.9 (9.7)**	0.39
Median (minimum, maximum)	34.0 (0, 76.0)	35.0 (0, 72.0)	**35.0 (0, 76.0)**	
**Sex**				
Female	88 (3.5)	46 (2.2)	**134 (2.9)**	<0.001
Male	2,415 (96.5)	2,064 (97.8)	**4,479 (97.1)**	
Missing data^†^	300	409	**709**	
**Race and ethnicity^§^**				
American Indian or Alaska Native	9 (0.3)	14 (0.6)	**23 (0.5)**	0.30
Asian	91 (3.5)	95 (4.1)	**186 (3.8)**	
Black or African American	580 (22.3)	546 (23.5)	**1,126 (22.8)**	
Hispanic or Latino	991 (38.1)	800 (34.4)	**1,791 (36.3)**	
Native Hawaiian or Pacific Islander	6 (0.2)	4 (0.2)	**10 (0.2)**	
White	809 (31.1)	787 (33.8)	**1,596 (32.4)**	
Multiple races	52 (2.0)	44 (1.9)	**96 (1.9)**	
Other race	66 (2.5)	35 (1.5)	**101 (2.0)**	
Missing data^†^	199	194	**393**	
**JYNNEOS vaccination status** ^¶^				
Fully vaccinated	166 (14.2)	128 (14.5)	**294 (14.3)**	<0.001
Partially vaccinated	134 (11.5)	62 (7)	**196 (9.6)**	
Unvaccinated	869 (74.3)	690 (78.4)	**1,559 (76.1)**	
Missing data^†^	1,634	1,639	**3,273**	
Death**				
Yes	5 (0.4)	1 (0.08)	**6 (0.2)**	<0.05
No	1,334 (88.0)	1,090 (88.0)	**2,424 (88.0)**	
Unknown**	175 (11.6)	148 (12.0)	**323 (11.7)**	
Missing data^†^	1,288	1,281	**2,569**	
**HIV status**				
Negative	438 (49.2)	404 (49.3)	**842 (49.2)**	0.10
Positive	394 (44.2)	327 (39.9)	**721 (42.2)**	
Unknown	59 (6.6)	88 (10.7)	**147 (8.6)**	
Missing data^†^	1,912	1,700	**3,612**	
**Hospitalized**				
Yes	239 (9.7)	201 (9.2)	**440 (9.5)**	0.77
No	2,053 (83.6)	1,856 (85.1)	**3,909 (84.3)**	
Unknown	162 (6.6)	124 (5.7)	**286 (6.2)**	
Missing data^†^	349	338	**687**	
**Sexual orientation**				
Bisexual	88 (9.7)	61 (8.9)	**149 (9.3)**	<0.001
Gay or lesbian	555 (61.2)	504 (73.3)	**1,059 (66.4)**	
Straight	130 (14.3)	64 (9.3)	**194 (12.2)**	
Different term	13 (1.4)	11 (1.6)	**24 (1.5)**	
Do not know	63 (6.9)	8 (1.2)	**71 (4.5)**	
Unknown	58 (6.4)	40 (5.8)	**98 (6.1)**	
Missing data^†^	1,896	1,831	**3,727**	
**Number of lesion locations**				
Two or fewer	406 (46.3)	206 (45.6)	**612 (46.0)**	0.29
Three to six	382 (43.6)	182 (40.3)	**564 (42.4)**	
Seven or more	89 (10.1)	64 (14.2)	**153 (11.5)**	
Missing data^†^	1,926	2,067	**3,993**	
**Lesion location^††^**				
Head or neck	305 (34.8)	165 (36.5)	**470 (35.4)**	<0.05
Face	250 (28.5)	155 (34.3)	**405 (30.5)**	
Mouth or oral mucosa	181 (20.6)	142 (31.4)	**323 (24.3)**	
Trunk	269 (30.7)	151 (33.4)	**420 (31.6)**	
Arms	313 (35.7)	180 (39.8)	**493 (37.0)**	
Hands	232 (26.5)	174 (38.5)	**406 (30.5)**	
Genitals	494 (56.3)	269 (59.5)	**763 (57.4)**	
Perianal region	224 (25.5)	118 (26.1)	**342 (25.7)**	
Legs	252 (28.7)	136 (30.0)	**388 (29.2)**	
Feet	70 (8.0)	50 (11.0)	**120 (9.0)**	
Other	308 (35.1)	103 (22.8)	**411 (30.9)**	
Missing data^†^	1,926	2,067	**3,993**	

* p-values compare differences across groups by year. For continuous variables, the Kruskal-Wallis test was used. Alternatively, for categorical variables, the chi-square test was used. However, if a variable was too sparse in data, Fisher’s exact test was used. All tests were two-sided (minimum reported as <0.001).

^†^ Missing is defined as information that was not reported in the data. Percentages do not include missing data in the denominator.

^§^ Persons of Hispanic or Latino (Hispanic) origin might be of any race but are categorized as Hispanic; all racial groups are non-Hispanic.

^¶^ Full vaccination was defined as receipt of 2 doses of JYNNEOS vaccine ≥14 days before illness onset and ≥24 days between the first and second dose. Estimates for partially vaccinated persons were unstable due to small numbers (no hospitalizations observed) and were excluded.

** All deaths have been confirmed by the jurisdiction as attributed to monkeypox virus, meaning monkeypox virus was listed on the death certificate as a cause of death or significant condition contributing to death.

^††^ These categories are not mutually exclusive.

### Vaccination Status

Vaccination status was reported for 2,049 (38.5%) patients over the 2-year period, with missing data increasing from 58% to 65%. In 2024, 166 (14.2%) patients were fully vaccinated, 134 (11.5%) were partially vaccinated, and 869 (74.3%) were unvaccinated. In 2025, 128 (14.5%) patients were fully vaccinated, 62 (7.0%) were partially vaccinated, and 690 (78.4%) were unvaccinated.

### Hospitalizations and Deaths

Hospitalization status was reported for 4,635 (87.0%) cases; among those, 9.5% of patients were hospitalized and hospitalization rates did not differ by year. Among patients with known vaccination status, 2.0% of vaccinated patients were hospitalized. In 2024, CFR was 0.18%, with five deaths reported among 2,803 patients with cases; two deaths occurred in unvaccinated patients and three in patients with unknown vaccination status. In 2025, CFR was 0.04%, with one death in an unvaccinated patient reported among 2,519 cases. Of 11 clade Ib cases, three patients were hospitalized and recovered without anti-orthopoxviral treatment; no clade Ib deaths were reported (*5*).

### MPXV Genomic Surveillance

Genomic surveillance in the United States during 2024–2025 revealed continued circulation of clade IIb, with approximately 97.5% of sequences belonging to descendants of 2022 outbreak lineage B** (Supplementary Table 1). Evidence of 21 importations was observed using case and viral genomic sequence data (available for 18 of 21 importations). Six MPXV sequences from the 11 clade Ib cases were linked to patients with travel from a clade Ib–affected country.^††^ Two clade Ib sequences were from a geographically limited domestic cluster reported during late 2025 (*4*). Twelve clade IIb lineage A.2.2 cases of MPXV infection were reported across eight states during 2024–2025. Ten of these patients reported travel to a country experiencing an outbreak caused by lineage A.2.2.^§§^ The remaining two A.2.2 sequences were associated with patients for whom travel history was unavailable and the associated sequences exceeded MPXV mutation rate estimations.^¶¶^ Together, the genomic and patient data for these 12 cases suggest importation events, rather than community transmission associated with lineage A.2.2 in the United States during 2024–2025.

### Multivariable Analysis

Among 860 (39%) U.S. cases of MPXV infection with available data, odds of hospitalization were 9.73 times as high among unvaccinated patients as among fully vaccinated patients, controlling for HIV status and age group (95% CI = 2.93–60.65) (Supplementary Table 2). The odds of hospitalization were 2.37 times as high among patients living with HIV as among patients without HIV, controlling for age group and vaccination status (95% CI = 1.52–3.74).

## Discussion

Persistent transmission of clade IIb MPXV in the United States, with wide geographic spread during the last 3 years, suggests a transition toward endemic circulation. The number of U.S. clade IIb cases of MPXV infection remained stable in 2024 and 2025; cases doubled during September–October 2025 even though the overall case counts remained lower than those reported during 2024. MPXV infection incidence among vaccinated patients was stable and consistent with expected vaccine performance given current vaccine effectiveness*** (*6*); coverage estimates do not suggest waning of vaccine-conferred immunity (*7*).

Detections of travel-associated cases of MPXV infection through a combination of genomic and epidemiologic surveillance data increased from 2024 to 2025. Demographic shifts observed in MPXV outbreaks globally prompted increased monitoring in the United States (Global mpox trends | World Health Organization). Several West African countries reported clade IIb lineage A.2.2 outbreaks characterized by similar proportions of cases among males and females, and evidence of heterosexual transmission (*8*). Within the United States, this pattern has been limited to travel-associated cases from countries where the A.2.2 lineage is circulating.

The United States reported 11 clade Ib cases during 2024–2025; however, no sustained transmission^†††^ was detected. Local transmission involving three cases in October 2025 was linked to a travel-associated introduction but did not result in further spread (*5*). Sustained transmission of clade Ib continues in several European countries, with approximately 89% of cases occurring among MSM (Global mpox trends | World Health Organization) (*9*). Clade Ib continues to spread among both MSM and heterosexual populations globally, with further importations into the United States observed in 2026 and expected in the future. Recommendations for treatment, infection prevention and control, vaccination, and case investigations remain unchanged, regardless of clade or subclade.

### Limitations

The findings in this report are subject to at least five limitations. First, mild infections for which care is not sought might not be captured by the passive MPXV surveillance system; therefore, the number of infections might be underestimated. Second, the reason for hospitalization is not a required field on the data collection form and is therefore not known for all cases; however, only hospitalizations related to MPXV infections were requested to be reported. Third, having virally unsuppressed HIV or a low CD4 T-cell count are known risk factors for hospitalizations related to MPXV infections, but these data are not collected on the short case report form. Had this variable been excluded from the model, the hospitalization rate would have been biased downward. Therefore, HIV was included as a binary variable with the assumption that it might not apply to everyone with HIV. Fourth, data collection methods vary by jurisdiction, including self-reporting or medical record verification. Finally, missing and unknown values were excluded for all data analyzed, which might lead to selection bias from complete case analysis (percentage missing = 62% [vaccination], 68% [HIV status], 70% [sexual orientation], and 75% [lesion location]).

### Implications for Public Health Practice

Continued circulation of clade IIb MPXV in the United States since 2022 suggests a transition toward endemicity. The changing epidemiology of MPXV infections globally, including new viral lineages and travel-associated cases, a doubling of cases during fall 2025 compared with fall 2024, and the higher odds of hospitalization among unvaccinated patients, all suggest that more work is needed to prevent MPXV transmission in the United States. CDC recommends continued surveillance to detect changing epidemiology and viral genomics, education for populations at risk for infection, and vaccination for populations at risk for infection (*10*).

## Footnotes

* Epidemiologic date was defined as the earliest date associated with a case (i.e., date of illness onset, diagnosis date, laboratory test date, or date reported).

† The population at risk for infection is estimated as MSM who have indications for HIV PrEP plus the number of MSM living with HIV. Because this estimate does not account for everyone at risk (e.g., women who had a male sexual partner who meets the previous criteria, MSM who do not have indications for HIV PrEP, or laboratory and health care workers working with orthopoxviruses), the population at risk is adjusted with an increase of 25% to account for the populations otherwise not included.

§ 45 C.F.R. Sect. 46.102(l)(2); 21 C.F.R. pt. 56; 42 U.S.C. Sect. 241(d); 5 U.S.C. Sect. 552a; 44 U.S.C. Sect. 3501 et seq.

¶ Travel history of cases linked to sequences from clade Ib or lineage A.2.2 were determined (when possible) by contact with the sequence submission authors or through publications linked to the sequence record. Viral lineage was determined using Nextclade.

** Overall, 81.5% of MPXV sequences descended from the 2022 outbreak (clade IIb, lineage B) in the United States during 2024–2025 belonged to the B.1.20 lineage (including B.1.20 and descendent F lineages). Other MPXV lineages in the United States were descendants of B.1.3 (including C and E lineages; 5.3%), sequences with no assigned sublineage usually due to missing data, short sequence length, or low quality (lineage B or B.1; 5.7%), B.1.22 and descendent J.1 (3.1%), B.1.21 (1.7%), and B.1.23 (0.1%).

†† Three of 11 clade Ib cases of MPXV infection were excluded due to missing sequence data (two) or collection date after December 31, 2025 (one).

§§ Reported travel dates occurred before symptom onset.

¶¶ Sequences from these two cases each had more than 15 single nucleotide mutations compared with other A.2.2 sequences from the same jurisdiction around the same 1- to 2-month span, which exceeds MPXV mutation rate estimates.

*** Vaccine effectiveness for MVA-BN in the general population is 35%–86% for 1 dose and 66%–90% for 2 doses.

††† Sustained transmission is defined as ongoing person-to-person transmission of MPXV with evidence that transmission is being maintained locally rather than resulting from sporadic importations or isolated transmission events.
